# Focus on the Breath: Brain Decoding Reveals Internal States of Attention During Meditation

**DOI:** 10.3389/fnhum.2020.00336

**Published:** 2020-08-28

**Authors:** Helen Y. Weng, Jarrod A. Lewis-Peacock, Frederick M. Hecht, Melina R. Uncapher, David A. Ziegler, Norman A. S. Farb, Veronica Goldman, Sasha Skinner, Larissa G. Duncan, Maria T. Chao, Adam Gazzaley

**Affiliations:** ^1^Osher Center for Integrative Medicine, University of California, San Francisco, San Francisco, CA, United States; ^2^Neuroscape Center, University of California, San Francisco, San Francisco, CA, United States; ^3^Department of Psychiatry, and Behavioral Sciences, University of California, San Francisco, San Francisco, CA, United States; ^4^Department of Psychology, University of Texas at Austin, Austin, TX, United States; ^5^Division of General Internal Medicine, University of California, San Francisco, San Francisco, CA, United States; ^6^Department of Psychology, University of Toronto, Mississauga, ON, Canada; ^7^School of Human Ecology and Center for Healthy Minds, University of Wisconsin-Madison, Madison, WI, United States

**Keywords:** meditation, interoception, attention, mind wandering, self-referential processing, multivoxel pattern analysis

## Abstract

Meditation practices are often used to cultivate interoception or internally-oriented attention to bodily sensations, which may improve health *via* cognitive and emotional regulation of bodily signals. However, it remains unclear how meditation impacts internal attention (IA) states due to lack of measurement tools that can objectively assess mental states during meditation practice itself, and produce time estimates of internal focus at individual or group levels. To address these measurement gaps, we tested the feasibility of applying multi-voxel pattern analysis (MVPA) to single-subject fMRI data to: (1) learn and recognize internal attentional states relevant for meditation during a directed IA task; and (2) decode or estimate the presence of those IA states during an independent meditation session. Within a mixed sample of experienced meditators and novice controls (*N* = 16), we first used MVPA to develop single-subject brain classifiers for five modes of attention during an IA task in which subjects were specifically instructed to engage in one of five states [i.e., meditation-related states: breath attention, mind wandering (MW), and self-referential processing, and control states: attention to feet and sounds]. Using standard cross-validation procedures, MVPA classifiers were trained in five of six IA blocks for each subject, and predictive accuracy was tested on the independent sixth block (iterated until all volumes were tested, *N* = 2,160). Across participants, all five IA states were significantly recognized well above chance (>41% vs. 20% chance). At the individual level, IA states were recognized in most participants (87.5%), suggesting that recognition of IA neural patterns may be generalizable for most participants, particularly experienced meditators. Next, for those who showed accurate IA neural patterns, the originally trained classifiers were applied to a separate meditation run (10-min) to make an inference about the percentage time engaged in each IA state (breath attention, MW, or self-referential processing). Preliminary group-level analyses demonstrated that during meditation practice, participants spent more time attending to breath compared to MW or self-referential processing. This paradigm established the feasibility of using MVPA classifiers to objectively assess mental states during meditation at the participant level, which holds promise for improved measurement of internal attention states cultivated by meditation.

## Introduction

Mind-body practices such as meditation are increasingly practiced by the public to improve health (Clarke et al., 2018), and train qualities of attention such as sustained focus, nonjudgment, and compassion to both internal and external stimuli (Kabat-Zinn, 2013; Gunaratana, 2010; Lutz et al., 2015). Through practicing these attentional qualities to internal bodily sensations such as the breath, meditation practices may strengthen interoception (awareness of internal bodily sensations; Farb et al., 2015; Khalsa et al., 2017), cognitive processes (including sustained attention, cognitive monitoring, and meta-awareness; Lutz et al., 2008; Dahl et al., 2015; Tang et al., 2015), and emotion regulation (less judgment and more equanimity with internal experiences; Chambers et al., 2009; Desbordes et al., 2015). With practice, these skills may lead to better monitoring and regulation of physical, emotional, and social processes, contributing to improved health decision-making and behaviors (Farb et al., 2015; Khalsa et al., 2017). However, interoceptive processes trained through meditation cannot be directly observed during practice because they are internal and fluctuate among various states of attention or inattention to the body (Van Dam et al., 2018). While previous studies assess neural activation during meditation to identify networks present at the aggregate level, currently no measure uses neural data to objectively assess whether attention is indeed focused on the body or not during meditation practice itself.

In other modalities of mental training, such as working memory training with external visual stimuli, the trained skills can be *directly observed and measured* in real-time with quantifiable metrics such as working memory performance (Klingberg, 2010). Also, subsequent *transfer effects* to other cognitive skills can be assessed (that are related but not directly trained), such as improvements in working memory in another sensory modality or inhibition (Klingberg, 2010). Thus, relationships between skills gained directly from training can be associated with transfer effects. Currently, interventions that train internally-oriented attention (such as meditation and yoga) have been studied mostly through transfer effects (i.e., downstream effects on external attention, emotion regulation, or well-being; Van Dam et al., 2018), largely because we lack measures that can objectively assess the focus of internal attention (IA) during the practice itself. The field, therefore, does not currently have a parallel measure to working memory performance, or metric of interoceptive focus or stability, that is both objective and unobtrusive to practice and could provide interoception metrics such as proportion time attending (or not) to the breath during meditation practice. With these metrics, we could more precisely understand how cultivating qualities of internal attention transfers to other psychological processes and more global states such as mental and physical health.

A measure that could directly assess the meditative process would be able to track various mental states as they fluctuate through time. For example, in a core practice of focused attention to the breath, attention is focused on sensations of the breath, until distracted by other internal or external stimuli, and then nonjudgmentally returned to the breath. Even in this simple practice, distinct mental states may occur and dynamically fluctuate over time: the object of attention (breath or distractions), level of meta-awareness (awareness of the object of attention), as well as attitudinal qualities such as nonjudgment, kindness, and curiosity (Hasenkamp et al., 2012; Lutz et al., 2015). Previous studies have mapped out neural networks associated with components of this process using fMRI, identifying greater activation in networks involved in interoception (Farb et al., 2013; Fox et al., 2016) and executive functioning (Brefczynski-Lewis et al., 2007; Fox et al., 2016), and decreased activation of the Default Mode Network (Brewer et al., 2011; Fox et al., 2016), which is engaged during mind-wandering and self-referential processing (Andrews-Hanna et al., 2014; Christoff et al., 2016). Although both states activate the Default Network, work by Christoff et al. (2016) conceptualizes mind wandering (MW) as distinct from self-referential processing, where MW can be characterized as movement from one mental state to another, which can include states of self-referential processing (such as engaging in self-related thinking about one’s life), creative thinking, or dreaming.

The traditional univariate fMRI analysis focuses on mean regional changes in brain activity and therefore usually requires the collapsing of data across many time points to improve signal estimation. The downside of this approach, particularly for the study of meditation, is that such data averaging obscures the fluctuating nature of distinct mental states, such as interoception, MW, and self-referential processing. These measurement issues can be addressed by instead applying multivariate multi-voxel pattern analysis (MVPA; Norman et al., 2006; Haxby, 2012), which uses pattern recognition technology to: (i) distinguish and recognize neural patterns associated with external or internal attention; and (ii) then apply these learned brain patterns to decode or estimate the presence of various mental states in a separate task. In this way, MVPA uses objective brain data to “read the mind” during tasks where the mental states are otherwise inaccessible (Norman et al., 2006; Haxby, 2012). For example, MVPA of fMRI data has been used to reliably differentiate the attentional status of two items held in working memory across an 8-s memory delay (Lewis-Peacock and Postle, 2012). On a trial-by-trial basis, these discrete neural measurements of internal attention have been linked to the precision of behavioral responses on short-term recognition tests (Emrich et al., 2013) and recognition confidence in tests of long-term memory (Lewis-Peacock and Norman, 2014).

In this proof-of-principle study, we aimed to produce quantifiable metrics of interoceptive attention by integrating MVPA methodology to study internal attention states relevant for meditation. We developed the EMBODY framework (**E**valuating **M**ultivariate Maps of **Body** Awareness), where MVPA is applied to neural data to: (i) learn and recognize internal attention (IA) states important for breath-focused meditation; and (ii) decode or estimate the presence of those IA states during an independent meditation period. We focused on three common IA states present during meditation—one state representing interoception (attention to breath) and two common distractor states from interoception (mind-wandering and self-referential processing). These decoded IA states could then be used to estimate the percentage time engaged in attention or inattention to the breath, providing an objective brain-derived metric of interoceptive focus during meditation.

## Materials and Methods

### General Framework and Approach

**Figure 1:**
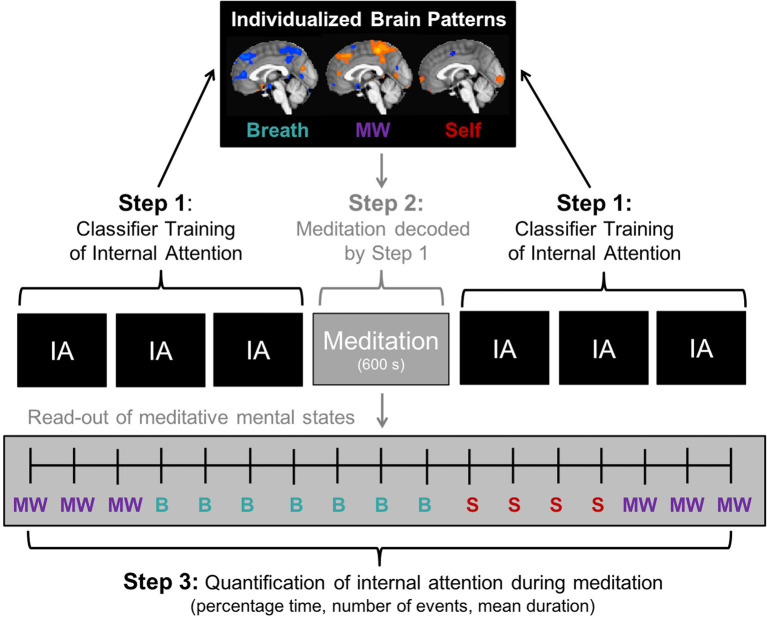
EMBODY Framework: **E**valuating **M**ultivariate Maps of **Body** Awareness to measure internal attention (IA) states during meditation. **Step 1**. Brain pattern classifier training. Machine learning algorithms are trained in fMRI neural patterns associated with internal mental states in the IA task. IA is directed *via* auditory instructions to pay attention with eyes closed to the breath, mind wandering (MW) self-referential processing (Self), and control conditions of attention to the feet and ambient sounds (see Figure 2). Individualized brain patterns for each participant are learned using n-1 cross-validation with six blocks of the IA task (volume *N* = 2,160). **Step 2**. Meditation period classification. Neural patterns are collected during a 10-min meditation period (in this case, focused attention to the breath; administered in the middle of six IA blocks), and are decoded by multi-voxel pattern analysis (MVPA) using the unique brain patterns learned in Step 1. Meditation is decoded second-by-second into mental states of attention to breath (B), MW, or self-referential processing (S), producing an estimate of distinct and fluctuating mental states during meditation. **Step 3**. Quantification of internal attention during meditation. From the temporal read-out of meditative mental states in Step 2, attention metrics during meditation can be quantified and estimated including percentage time spent in each mental state, the number of times engaged in each mental state (“events”), and mean duration spent in each mental state.

**Figure 2:**
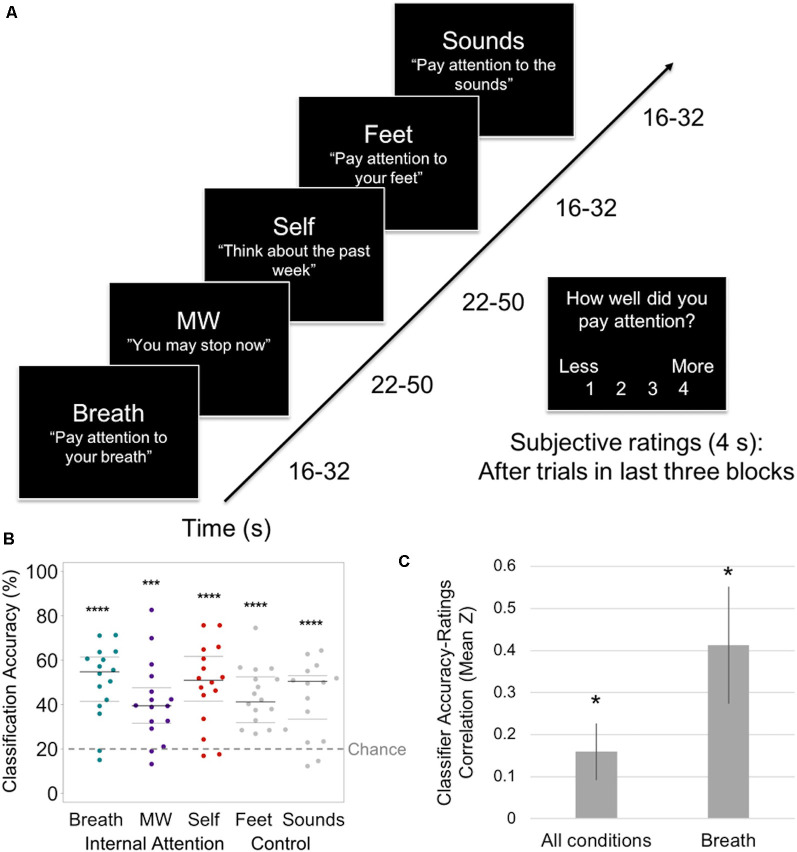
EMBODY Step 1: classifier training of internal mental states. **(A)** Internal Attention (IA) task. With eyes closed, participants were directed *via* 2-s auditory instructions to pay attention to five internal mental states for brief periods (16–50 s). The IA task-directed attention to three mental states relevant for breath meditation [Breath, mind wandering (MW), and Self], and two control mental states [attention to the Feet (another area of the body) and ambient MRI Sounds (consistent external distractor)]. The example auditory instructions are displayed in quotes. MW was induced by instructing participants to stop paying attention and let their minds attend to whatever they wanted. Conditions were randomized over six IA blocks in four orders, with 72 s of data collected from each condition in each block (total 432 s/condition). For the last half of IA task trials, subjective ratings of attention were collected after each trial (except MW) using a button box (1 = less, 4 = more). **(B)** From the IA task, the prediction accuracy of the classifier for identifying internal states of attending to the Breath, MW, and Self, and control conditions of attending to the Feet and Sounds. Beeswarm plots present each data point, the median (bold black line), and ±25th percentile range (gray lines) of the mean prediction accuracy for all data in each condition (*n* = 432) across all subjects. Statistical significance was determined by a one-sample two-sided *t*-test against theoretical chance-level for classification of five categories (20%, denoted by a dashed line). ***t_(15)_ = 4.65, *p* < 0.001, ****ts_15_ > 5.67, *p*s < 0.0001. **(C)** Mean *z*-scores representing the within-subject correlation between trial-level classifier training accuracy and subjective ratings of attention (administered during the last half of IA task trials) for all conditions (except MW) and breath trials only. Error bars indicate standard error of the mean. *p < 0.05.

We tested the feasibility of the EMBODY framework within 16 participants in three steps, where MVPA is applied to fMRI data to learn and decode mental states during meditation, producing novel individual-level metrics of internal attention during meditation (Figure 1). In Step 1, at a participant-specific level, we first tested whether IA brain patterns (breath, MW, self-referential processing) could be reliably learned and distinguished above chance levels by MVPA classifiers. In a directed Internal Attention task, participants were specifically instructed to engage in one of five states (i.e., meditation-related states: breath attention, MW, and self-referential processing, and control states: attention to feet and sounds; Figure 2A), and MVPA was applied to develop single-subject brain classifiers for five modes of attention. Using standard cross-validation procedures, MVPA classifiers were trained in five of six IA blocks for each subject, and predictive accuracy was tested on the independent sixth block (iterated until all block volumes were tested, *N* = 2,160).

Notably, MVPA is often applied using a within-subjects approach, with more data collected from each person so that MVPA classifiers can learn and decode brain patterns that are participant-specific (Norman et al., 2006; Haxby, 2012). Similar to what is commonly done in the visual sciences, this approach concentrates experimental power and high-powered tests of effects at the individual level (Smith and Little, 2018), and the generalizability of the findings can be assessed by examining the proportion of the subjects in which MVPA is reliable. To maximize this within-subjects approach, we collected more data within each participant (2,160 brain patterns for classifier training and testing, 432/condition). We tested this framework in eight meditators and eight controls (an adequate *N* for within-subjects MVPA studies; Norman et al., 2006) and included individuals from both groups because: (a) meditators are more likely to produce distinct brain patterns from consistent practice in directing and sustaining internal attention; and (b) novices are the population most studied in clinical intervention studies. To further test whether MVPA classifier accuracy of internal attention was a neural marker of better attention, we associated accuracy with within-subject subjective ratings of attention.

By first establishing that MVPA classifiers could reliably distinguish and identify internal attention brain states in Step 1, we could then apply these learned brain patterns in Step 2 to objectively decode or classify the continuous focus of attention using neural data from a separate 10-min meditation period (600 novel brain patterns). This is a common application of MVPA, where classifiers learn neural patterns from distinct categories in one task (and classifier accuracy is validated using within-task cross-validation), which are then used to estimate information in a separate task (across-task decoding where classifier training from the first task is used to inform classifier decisions in the second task; Norman et al., 2006), and the EMBODY framework extends this approach into decoding IA states during meditation. In Step 3, we used these classified IA states to make an inference about attention states during meditation, including percentage time engaged in breath-focus, MW, or self-referential processing. This thus served our main measurement goal of *estimating the interoceptive focus or stability* on the breath during meditation practice for each individual. Finally, to assess construct validity and inform future research, we preliminarily characterized the meditation metrics at the group level and tested whether participants attended longer to breathe vs. other mental states during meditation.

### Participants

Participants were medically and psychiatrically healthy adults age 25–65, non-smokers, and MRI-compatible. Meditators were recruited from Bay Area meditation centers through flyers, online postings, and word of mouth. They reported consistent meditation practice in the past 5 years (≥90 min/week, ≥14 days of silent retreat practice, at least half of practice on breath and bodily sensations). Control participants were recruited through flyers and online postings, and had not engaged in regular meditation or other mind-body practices (>20 min at least twice weekly), and were age (within 5 years) and gender-matched to meditators.

Participants included eight meditators [one female, one non-binary person, six male, mean age = 38.4 (range 28–61), race/ethnicity: six White, two multiracial] and eight novice control participants matched in gender and age [mean age = 38.3 (range 25–63), race/ethnicity: six White, one Asian, one Latinx/Hispanic; see Supplementary Table S1 in Supplementary Information [SI] for full demographics]. The average lifetime meditation practice in meditators was 3,495 h (range 509–6,590; Supplementary Table S2). Two additional novices were excluded for the inability to align images due to excessive movement and incorrect gender-matching to a meditator.

### Procedure

Eligibility was assessed by an online questionnaire and phone interview. Participants were consented, trained in MRI task procedures, and then completed a 2-h MRI protocol. They were paid $65 for participation and ≤$20 for travel. All participants provided written informed consent approved by the Institutional Review Board of the University of California, San Francisco. The study was registered at www.clinicaltrials.gov, identifier NCT03344081.

### fMRI Paradigm

#### Overall Framework

The EMBODY Framework used (MVPA; Norman et al., 2006) with fMRI data to decode the focus of internal attention during meditation in three steps: (1) participant-specific brain patterns were trained for internal attention states relevant for breath meditation (*N* = 2,160); (2) brain patterns learned from Step 1 were applied to a 10-min period of breath meditation to estimate the focus of internal attention for each data point (600 s); and (3) metrics of attention during meditation were computed from the decoded brain states (Figure 1).

#### Step 1 Data: Internal Attention (IA) Task

fMRI data from the IA task were used to train a machine learning classifier to learn neural patterns associated with five internal mental states. To create training data that closely resembled brain activity during meditation, participants’ eyes remained closed, and the only stimulus change was their internal focus of attention. Neural patterns associated with breath, MW, and self-referential processing was chosen to be most relevant for decoding the meditation period, which modeled the intended interoceptive focus of meditation (breath) and two common distractors from interoception (mind-wandering and self-referential processing). Neural patterns associated with attention to feet (interoception to another body area) and awareness of ambient sounds (consistent MRI sounds) were chosen as control conditions to improve the classification specificity of the desired brain states. Mind-wandering and self-referential processing were treated as distinct mental states based on the Christoff et al.’s (2016) conceptualization of MW as movement from one mental state to another, and self-referential processing as a common mental state MW can shift towards. Also, self-referential processing is an important mental state to isolate because of contemplative theories that emphasize the benefits of becoming aware of and de-identifying with self-related thought (Dahl et al., 2015). Classifier specificity of attention to the breath is improved by including attention to feet (another area of the body), and classifier specificity of all internal conditions is improved by including a common external distractor—ambient sounds in the MRI scanner.

Participants received randomized 2-s auditory instructions to pay attention to: (1) sensations of the breath (Breath); (2) sensations of the feet (Feet); (3) to stop paying attention and let their minds go wherever they would like (mind wandering or MW); (4) self-referential processing regarding the past, present, and future (Self); and (5) ambient sounds (Sounds; Figure 2A). For breath-focus, attention was maintained where they felt the breath most strongly (e.g., nose, throat, chest). For self-referential processing, participants generated five events from the past week and five events that would occur in the next week during the training session. They were also instructed to engage in self-referential processing regarding the present moment (“Think about right now”), and think about their experience during the experiment. Instructions were also presented visually, which participants could briefly view as a reminder (no participants reported using this strategy). Six blocks were administered from one of four randomized stimulus order sets.

Each of six blocks contained 20 s of baseline (black screen) at the beginning and the end and consisted of 13 trials/block. These consisted of three trials each for Breath, Feet, and Sound conditions (ranged from 16–32 s), and two trials each for MW and Self conditions (ranged from 22–50 s). Trial durations for attending to breath, feet, and sounds were shorter in length (16–32 s; three trials/block each) to increase the likelihood that participants could maintain stable attention and produce a consistent neural signal. Every even-numbered trial length was randomized and administered twice/condition across the experiment. Trial durations for MW and Self were made longer (22–50 s; two trials/block each) to allow more time for these states to occur (as longer scan times as in resting-state studies are used to induce MW and self-referential processing; Andrews-Hanna et al., 2014). Trial lengths covered most of the duration range across the experiment. This protocol resulted in a balanced design of 72 s/condition within each block and yielded 432 patterns/condition and 2,160 total training brain patterns over the entire experiment. In the last three IA blocks, participants subjectively rated how well they paid attention after each trial using a button box (*How well did you pay attention?* 1 = less attention, 4 = more attention), and were encouraged to use the full range of responses.

#### Step 2 Data: Meditation Period

Participants engaged in 10 min of breath-focused meditation in two blocks, administered between the six IA blocks. The meditation period was split into two blocks (4 and 6 min) to help control participants stay engaged in the task. Participants were instructed to pay attention to sensations of the breath, and if their minds wandered, to return attention to the breath. For each block, they received a 6-s instruction at the beginning, and a 2-s reminder to pay attention 60 s before the end. After the meditation period, participants verbally rated the percentage time they paid attention to the breath and thoughts for each block.

### Data Acquisition

Experiments were run using E-Prime (Psychology Software Tools). Neuroimaging data were acquired with a 3T MRI scanner (Siemens Prisma) using a 64-channel head coil. A high-resolution 1 × 1 × 1 mm MPRAGE T1 anatomical scan was acquired for offline spatial registration. Functional images were acquired using a multiband gradient-echo EPI sequence (2.4 × 2.4 × 2.4 mm, TE/TR = 30.2 ms/1 s, FOV = 220 mm, 92 × 92 matrix, 56 slices, multiband acceleration = 4, TR = 1.0 s; Auerbach et al., 2013) that covered most of the brain.

### EMBODY fMRI Data Analyses: Machine Learning

#### fMRI Preprocessing

Data were preprocessed in AFNI (Cox, 1996) and were slice time corrected, aligned and motion-corrected to the first volume of the first EPI scan, and linearly de-trended in native space, respectively using 3dTshift, 3dAllineate, 3dvolreg, 3dDetrend. See Supplementary Information (SI) p. 2–3 and Supplementary Table S3 for control analyses using head motion and respiration data.

#### Step 1 Machine Learning: Distinguishing Neural Patterns of Internal Attention (Within-task Cross-validation)

Pattern classification analyses were conducted using MVPA (Norman et al., 2006; Princeton MVPA Toolbox^1^), in conjunction with in-house software using Matlab (MathWorks) and Python (for post-processing of meditation period classifications in Steps 2–3). Using whole-brain preprocessed fMRI signal in native space, a pattern classifier was trained separately for each participant for trial periods from each condition (Breath, MW, Self, Feet, and Sounds; TR = 1.0 s, 432 s/condition) using penalized logistic regression with L2 regularization and a penalty parameter of 0.01 (which prevents over-fitting by punishing large weights during classifier training; Duda et al., 2000). Condition labels were shifted in time by 6 s to account for hemodynamic lag. A binary logistic regression (1 vs. the others) was run for each of the five conditions, resulting in continuous classifier evidence values (0–1) for each condition at each time point in the experiment. The condition that was assigned the highest evidence value yielded the categorical decision from the classifier (see Supplementary Information (SI) p. 4 and Supplementary Table S4 for alternate analyses of classifier evidence and decisions). We evaluated classification accuracy by performing k-fold cross-validation analysis, training on five blocks of data (fMRI task runs), and testing on the novel sixth block. The training blocks were then rotated, and a new block of data was tested until all six blocks of data had been classified (2,160 decisions).

Classification accuracy for each condition was computed for each participant (the percentage out of 432 accurate decisions output by the machine learning classifier). Group-level accuracy for each condition was tested with a one-sample *t*-test vs. 20% (theoretical chance level for five conditions), and the effect size was estimated with Cohen’s *d*. Individual-level accuracy was tested with a Chi-square test determining whether the number of accurate vs. inaccurate decisions in each condition were significantly above chance distribution (87 vs. 345). Individuals that showed above-chance accuracy in 2/3 categories for Breath, MW, and Self conditions were used for subsequent analyses including decoding meditation states (all eight meditators and 6/8 controls).

#### IA Ratings and Classifier Accuracy

Attention ratings were collected in the last three blocks, which included 33/39 trials. The remaining six MW trials were excluded because participants were instructed to not pay attention, so no rating was administered. To estimate the correlation of classifier accuracy and ratings within each subject, Pearson’s correlation was computed between trial-level classifier accuracy and subjective ratings of attention. To test the strength of correlations across the group, each individual *r*-value was transformed using a Fisher *r*-to-*Z* transform, and the group mean *Z*-score was tested vs. 0 using a one-sample *t*-test. Because this task was designed to measure breath attention, we also examined accuracy-rating correlations specifically in Breath trials (*N* = 9).

#### Individualized Brain Pattern Importance Maps

Classifier importance maps were computed for each participant using classifier weight information which identifies which voxels were most important in distinguishing neural patterns of Breath, MW, and Self (McDuff et al., 2009). To identify voxels that distinguished between conditions that were not due to differences in head motion, the analyses were conducted with fMRI data where motion variables were covaried out. We identified voxels with “positive importance” (both the weight and *z*-scored average activation value are positive) and voxels with “negative importance” (both the weight and *z*-scored average activation value are negative). Note that this approach identifies voxels which aid classifier distinction between mental states, and does not test for differences in an average activity like standard univariate analyses (Haxby, 2012). For display purposes, each individual’s importance maps were non-linearly warped to the MNI152 2 mm template using FSL (FNIRT; Smith et al., 2004), smoothed with an 8 mm Gaussian kernel, converted to *z*-scores (across voxels), and thresholded at ±2 SD to identify the most important voxels for each condition. To characterize whether importance voxels were present within cortical and subcortical regions at the individual level, we created general masks from the 2-mm Harvard-Oxford cortical and subcortical atlases from FSL (0% gray matter thresholding to be as inclusive as possible). All regions from each atlas were included in the general masks, except for Cerebral Cortex and Cerebral White Matter regions from the subcortical atlas (which included large cortical regions). The percentage of importance voxels found within the cortical and subcortical masks were computed for each subject and summarized at the group level with mean and SD.

#### Step 2 Machine Learning: Decoding the Internal Focus of Attention During Breath Meditation (Across-task Decoding)

Individualized brain patterns learned from Step 1 were applied to the 10-min meditation period to decode the internal focus of attention. The meditation period consisted of a completely independent dataset, and these classifiers were not influenced in any way by the previous Step 1 cross-validation analyses. The classifier was trained with all five mental states from the IA task (2,160 total brain patterns) and decoded with the three states that were most relevant for breath-focused meditation: Breath, MW, and Self. For each data point during meditation (*N* = 600, excluding instruction periods), the classifier output a categorical decision of whether the internal focus was on the Breath, MW, or Self (as well as continuous evidence values for each mental state). This produced a continuous estimate of mental states during meditation over time.

To ignore spurious measurements of brain states that may fluctuate from one-time point to the next, we focused our analyses on relatively stable periods. We defined a “mental event” as the classification of three or more consecutive time points for a given category. To facilitate this, we smoothed the data such that a single incongruous decision between two events of the same type (e.g., MW event–Self-decision–MW event) were relabeled according to the category of the surrounding events (e.g., Self => MW; average data points smoothed = 1.3%, SD = 0.41). Events were then quantified as three or more consecutive decisions of the same category, excluding any data that did not meet these criteria (average data excluded = 15.7%, SD = 4.82). See Supplementary Information (SI) p. 4 and Supplementary Table S4 for additional analyses with no smoothing function and varying mental event lengths (2 and 4 s).

#### Step 3: Quantify Internal Attention Metrics During Meditation

From the mental state classifications from Step 2, novel metrics of internal attention during meditation were computed for each participant. For each mental state, *percentage time engaged*, *the number of events*, *mean duration of events*, and *variability* (SD) of event duration was computed. Data were preliminarily analyzed at the group level by testing for differences in metrics between conditions (Breath, MW, Self) with a one-way ANOVA. To test our main hypotheses that breath-focused meditation would result in differences between Breath vs. other mental states, significant results were followed up with planned pair-wise *t*-tests of Breath vs. MW and Breath vs. Self. Data were analyzed in SPSS (v. 24), figures were created with R, and brain maps were displayed using AFNI or FSLview.

## Results

### Step 1: Distinguishing Neural Patterns of Internal Attention

The first aim of the EMBODY framework was to test whether MVPA applied to within-subject fMRI data could recognize individualized neural patterns associated with internal attention states important for breath meditation (Figure 2A). Across all participants, each attentional state yielded a distinct neural signature (all classification accuracies >41% vs. 20% chance for five categories, *p*s < 0.001; Figure 2B). Furthermore, each attentional state was distinguished at more than twice chance levels, including the brain patterns most relevant for breath meditation (breath = 50.5%, MW = 41.2%, self-referential processing = 49.0%; *t*s_(15)_ > 4.65, *p*s < 0.001, Cohen’s *d*s > 1.16) and the control conditions (feet = 43.2%, sounds = 43.7%, *t*s_(15)_ > 5.67, *p*s < 0.0001, Cohen’s *d*s > 1.41; Figure 2B; see Supplementary Table S5 for the classifier confusion matrix).

The breath meditation-relevant brain patterns were reliably classified in 14/16 or 87.5% participants (at least 2/3 *p*s < 0.001 for breath, MW, and self-referential processing; Supplementary Table S6). This included all eight meditators and 6 of 8 novices, all of whom were used in subsequent analyses. Within-subject correlations of trial-level classification accuracy and subjective ratings of internal attention (across all conditions except MW) showed a positive association overall across the group (mean *Z* = 0.16, one-sample *t*_(13)_ = 2.35, *p* = 0.035; Figure 2C). Because the task was designed to specifically measure breath attention during meditation, we also examined associations between accuracy and ratings in breath-trials only and found a higher mean correlation (mean *Z* = 0.41, *t*_(13)_ = 2.96, *p* = 0.011; Figure 2C).

### Distributed Brain Patterns Contributing to Accurate IA Classification

**Figure 3:**
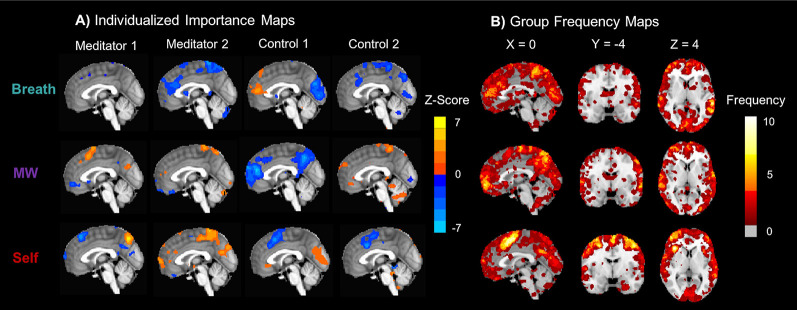
Classifier importance maps representing voxels that accurately distinguish internal mental states. **(A)** Subject-level importance maps showing individualized brain patterns representing voxels that are important for distinguishing neural signatures of attention to the Breath, MW, and Self (*X* = 0). For each task condition, importance values were computed by multiplying each voxel’s classifier weight for predicting the condition and the average activation during the condition (McDuff et al., 2009). The maps were thresholded at ±2 SD and displayed on the MNI152 template to identify the most important voxels for each participant. Orange importance voxel indicates positive *z*-scored average activation values, and blue importance voxels indicate negative *z*-scored average activation values. **(B)** To initially characterize the distribution of voxels that supported accurate classification, group importance frequency maps indicate the number of participants for which the voxel accurately distinguished each mental state. All important voxels were summed, irrespective of average positive or negative *z*-scored activation. Frequency maps were also computed that independently summed positive (Supplementary Figure S2A) and negative (Supplementary Figure S2B) *z*-scored activation voxels, as well as histograms of frequency counts (Supplementary Figures S2C–E). Note that the maximum frequency for any importance map was 10/14.

Classifier importance maps identified the voxels most important in distinguishing between the attentional states (McDuff et al., 2009), which were distributed throughout the brain and unique for each participant (Figure 3A). The majority of voxels that contributed to accurate classification were distributed throughout cortical regions [Breath mean = 93.6% (6.2), MW mean = 92.9% (5.5), Self mean = 93.7% (5.6)], with fewer located in subcortical areas [Breath mean = 1.4% (1.6), MW mean = 1.4% (1.4), Self mean = 0.72% (1.1)]. For the initial characterization of the distribution of voxels that supported classification, a frequency map was computed representing the sum of individual importance maps (Figure 3B). This indicated that no brain region was important for all 14 participants in any mental state (maximum frequency ≤10; Figure 3B, Supplementary Figures S2A,B), and frequency histograms showed that most voxels were important for only 1–3 participants (Supplementary Figures S2C–E).

### Step 2: Decoding the Focus of Attention During Breath Meditation

**Figure 4:**
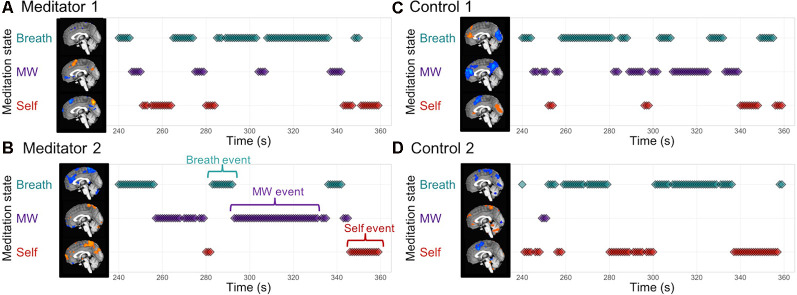
EMBODY Step 2: decoding the internal focus of attention during breath-focused meditation using individualized brain patterns. Based on each participant’s unique brain signatures for Breath, MW, and Self, classifier decisions were made for each time point of fMRI data (TR = 1 s), producing a continuous estimate of attention states during a 10-min breath meditation. The middle of the meditation period is displayed for two meditators **(A,B)** and their matched controls **(C,D)**. Mental events were quantified as three or more consecutive decisions from the same mental state **(B)** and were used to compute metrics of attention during meditation in Step 3. See “Materials and Methods” section and Supplementary Information (SI) p. 4 and Supplementary Table S4 for details and alternate data reduction of classifier evidence and decisions.

Individualized brain patterns for each participant were used to decode the focus of attention during 10 min of breath meditation, producing a continuous estimate of internal attention states of attending to the breath, MW, or self-referential processing (Figures 4A–D). Classifier decisions at each time point were based on the class with the highest classifier evidence values (Supplementary Figure S2). From these data, “mental events” were defined whenever there were three or more consecutive time points that were classified as belonging to the same mental state (Figure 4B). See Supplementary Information (SI) p. 4 and Supplementary Table S4 for additional analyses with alternate data reduction of classifier evidence, decisions, and mental event length.

### Step 3: Quantifying Metrics of Internal Attention During Breath Meditation

Based on MVPA classification of mental states during meditation from Step 2, we computed metrics of attention during meditation for each participant, including *percentage time spent engaged* in each mental state, the *number of mental events* (or discrete periods engaged in each mental state), the *duration of each mental event*, and the *variance of the durations* (SD).

### Attention Profiles During Breath-Focused Meditation

**Figure 5:**
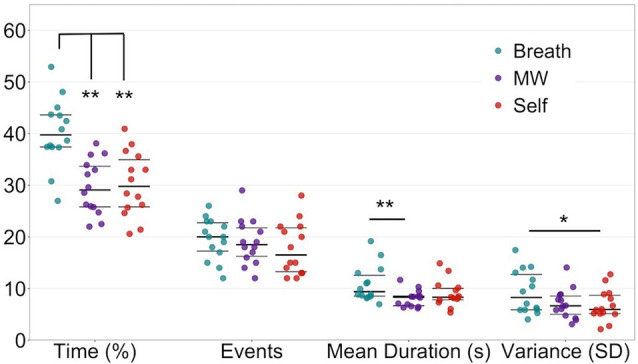
EMBODY Step 3: quantification and mental state profiles of internal attention during meditation. Based on the estimate of mental states and event specification from Step 2, metrics of attention during breath meditation were quantified for each mental state and initially characterized at the group level: percentage time spent in each mental state (Breath, MW, or Self), the number of events, mean duration of events (s), and variability (standard deviation or SD) of the duration of events. Overall, participants spent more time attending to the breath vs. MW or self-referential processing. Beeswarm plots present each data point, the median (bold black line), and ±25th percentile range (gray lines). See Supplementary Table S7 for full metric statistics. *paired *t*_(13)_ = 2.46, *p* = 0.029, after one-way ANOVA *F*_(2,12)_ = 4.20, *p* = 0.026. **paired *t*s_(13)_ ≥ 3.18, *p*s ≤ 0.007, after one-way ANOVA *F*s_(2,12)_ ≥ 6.47, *p*s ≤ 0.005.

Although the main goal of this study was to test the feasibility of using MVPA to estimate IA states during meditation at the individual level, we preliminarily characterized attention profiles at the group-averaged level (Figure 5; Supplementary Table S7). For breath-focused meditation, we hypothesized that participants would direct their attention more to the breath than engaging in MW or self-referential processing. Therefore, compared to the other mental states, participants should show greater: (1) percentage time attending to the breath; (2) the number of breath mental events; (3) mean duration of attention to the breath; and (4) variance in duration on the breath (greater inter-trial variability due to longer durations). Attention metrics differed in the percentage time engaged in each mental state (*F*_(2,12)_ = 8.93, *p* = 0.001), the mean duration of mental events (*F*_(2,12)_ = 6.47, *p* = 0.005), and the mean-variance of event durations (*F*_(2,12)_ = 4.20, *p* = 0.026). Consistent with our hypotheses, we found that during meditation, participants spent more time paying attention to their breath compared to MW or self-referential processing (*ts*_(13)_ > 3.18, *p*s < 0.007). These results remained consistent even with alternate data reduction of classifier decisions, evidence, and event length during the meditation period [see Supplementary Information (SI) p. 4 and Supplementary Table S4 for full details].

On average, the 10-min meditation periods contained 56.4 mental events of at least 6-s (SD = 11.26). Although the mean number of events across mental states did not differ significantly (*p* = 0.31), when participants attended to the breath, the mean duration of those events [10.9 s (3.5)] was longer than for mind-wandering events [8.1 s (1.6), *t*_(13)_ = 3.28, *p* = 0.006] and marginally longer than self-referential processing events [9.0 s (2.6), *t*_(13)_ = 1.94, *p* = 0.07]. Similarly, the variability of event durations tended to be greater for attention to the breath compared to both MW (*t*_(13)_ = 1.92, *p* = 0.08) and self-referential processing (*t*_(13)_ = 2.46, *p* = 0.029). See Supplementary Table S7 for full statistics (including p. 6 for a distraction from breath and mental state fluctuations). See Supplementary Information (SI) p. 6–8 for preliminary correlations between attention metrics and between-subject variables [self-reported attention during meditation, lifetime meditation practice (Supplementary Figure S3) and trait interoception and mindfulness].

## Discussion

This proof-of-principle study tested the feasibility of the EMBODY framework, where MVPA pattern classifiers were applied to neural data to learn and decode internal attention states during meditation, producing novel estimates of interoceptive focus during meditation. We demonstrated that fMRI pattern classifiers could indeed distinguish between five states of internal attention using participant-specific brain patterns. This analysis was successful in all but two participants (87.5%; including all eight experienced meditators and 6/8 novice controls), demonstrating that MVPA recognition of directed IA states has high generalizability, particularly for experienced meditators. Further, within-subject classification accuracy was positively correlated with subjective ratings of internal attention, suggesting that the EMBODY framework can reliably assess neural patterns representing internal attention. These neural patterns were then used to continuously decode the presence of breath attention, MW, and self-referential processing during an independent 10-min period of breath-focused meditation. By making these invisible internal processes visible and quantifiable, we were able to compute novel profiles of attention during meditation, including the percentage of time engaged in breath attention, MW, or self-referential processing (as well as the number of mental events, mean duration, and variance). Preliminarily, across all participants with distinguishable brain patterns, attention profiles indicated they engaged more with the breath vs. other states (greater percentage of time attending to the breath and greater mean duration of breath events). This objective measure provides initial evidence that participants were able to implement the meditative goal of sustaining attention to the breath. Together, these findings support the feasibility of employing the EMBODY framework to utilize participant-specific neural data to estimate interoceptive focus to the breath and other mental states during meditation.

To establish reliable neural patterns to decode meditation states, we first tested whether directed internal attention states could indeed be recognized by MVPA in the IA task. Interestingly, even with no changes in the external visual environment, by simply directing the internal focus of attention to five types of internal stimuli (interoceptive sensations of the breath and feet, engaging in MW or self-referential processing, and listening to ambient sounds), this produced reliable and distinct neural patterns for most participants. Further, the initial construct validity of the IA task was supported through evidence that within-subject neural classification accuracy (which indicates reliability and distinctiveness) was positively correlated with subjective attention ratings, particularly for breath trials. This may suggest that more consistent IA neural patterns may contribute to a better subjective sense of IA; however, this relationship should be tested further with more trials in a larger sample size. This approach worked in all experienced meditators and most novice controls, suggesting that the task may be applied in both cross-sectional and longitudinal study designs. However, the IA task needs further validation in larger samples, and should be improved to increase classifier performance (optimizing trial conditions and durations, using feature selection with functional networks, testing different classification algorithms, integrating psychophysiological and behavioral data), and use real-time neurofeedback to aid interoceptive focus (Sitaram et al., 2017). These early results motivate this future work and support the feasibility of using MVPA to distinguish between different internal attention states using a participant-specific approach.

Notably, the important voxels that contributed to accurate classification for each mental state were distributed across many areas of the brain and tended to be unique for each participant, which lends support to using individualized MVPA approaches to measure IA states. The majority of these important voxels were located in cortical rather than subcortical regions. Further research with more subjects can begin to characterize which networks in the cortex may be most contributing to accurate classification, including interoception, executive functioning, and DMN networks. Similar to previous research (Kerr et al., 2011), these results demonstrated that neural signals may differentiate interoception to distinct areas of the body (breath vs. feet), which could potentially track attention during body scan practices. These findings also showed that the brain patterns for MW, or the “movement” from one mental state to another (Christoff et al., 2016), were distinct from self-referential processing, which demonstrated that the wandering nature of attention could be disentangled from the *contents* of what the mind wanders to. The neural pattern for mind-wandering may also contain a conglomeration of mental states, some of which may be MW or mental movement, and other potential states such as creative thinking (Christoff et al., 2016) or attention to other internal or external stimuli that were not included in the IA task. Further research may include more mental states to accurately identify and classify MW.

By establishing that MVPA could recognize distinct IA states, we could then apply these classifiers to estimate the presence of IA states using neural patterns during a separate meditation period. We demonstrated the feasibility of using neural patterns to estimate mental states during meditation, producing a temporal read-out of mental states that could be used to estimate the percentage time engaged in interoceptive focus. To our knowledge, this study provided the first objective measure that enabled continuous estimation of mental states that did not impact the meditative process by requiring subjective or motor responses (Levinson et al., 2014). However, MVPA decisions of meditative mental states should be further validated with participant subjective and behavioral responses of what mental states are present during meditation (Hasenkamp et al., 2012) and associations with other measures of interoception and mindfulness in larger samples. Although the focus of the current approach was on within-subject analyses, we preliminarily assessed differences in attention profiles across participants and found that participants spent more time attending to the breath vs. MW or self-referential processing during meditation (even with alternate analyses of classifier evidence and decisions), likely due to the longer duration on the breath when attention was directed there. This early result suggests that attention may be reliably directed towards a focused internal stimulus such as the breath; however, this should be replicated in studies with larger sample sizes, and meditative attention profiles should be compared to other states such as resting state. Although in these initial results, attention to the breath was greater compared to the other mental states individually, if these non-breath mental states are combined, their total time is greater than attention to the breath. To clarify how attention to the breath is modulated by meditation, it would be important to establish baseline attention levels during a resting state scan and examine how all three mental states change from baseline to meditation (particularly in experienced meditators vs. controls).

Further, future research may investigate alternate ways to analyze the meditation period using classifier evidence, which can represent mental states as simultaneously occurring that wax and wane at different rates (compared to classifier decisions that represent single states that categorically shift over time). With larger samples, researchers can investigate whether more nuanced metrics using classifier evidence may be meaningfully related to other measures of interoception, mindfulness, and clinical outcomes. Additionally, given the high cost and limited temporal resolution of fMRI-based measures, the framework should be extended using alternative neuroimaging methods such as electroencephalography and magnetoencephalography (Zhigalov et al., 2019).

Overall, the initial EMBODY framework shows promising ability to distinguish unique brain patterns of internal attention, which can then be used to estimate mental states during meditation, particularly interoceptive focus to the breath. These new metrics may aid the measurement of internal focus during meditation practice, which could elucidate how cultivating qualities of internal attention may transfer to cognitive and emotion regulation. Given that meditation trains multifaceted qualities of attention, the framework may be adapted to measure other aspects of attention (e.g., meta-awareness, nonjudgment) and meditation practices (e.g., open monitoring, compassion). By developing measures to precisely assess the attentional qualities cultivated by meditation, we will gain the measurement power needed to rigorously test the attentional and emotional mechanisms through which meditation may improve health and well-being. Finally, the EMBODY framework highlights that each individual’s brain signatures and meditation practice are unique, which we hope will aid researchers and clinicians in designing interventions that will maximally benefit individuals in targeted and specific ways.

## Data Availability Statement

Code for the EMBODY Task, MVPA analysis, and post-processing are available at https://github.com/HelenWengPhD/embodystudy. MRI data are available from participants who consented to share raw brain data (links at Github page).

## Ethics Statement

The studies involving human participants were reviewed and approved by Institutional Review Board of the University of California, San Francisco. The patients/participants provided their written informed consent to participate in this study.

## Author Contributions

HW, JL-P, FH, and NF conceptualized the study. All authors contributed to the data analytic strategy and interpretation. HW, SS, and VG contributed to data collection, processing, and analysis. HW, JL-P, SS, and VG developed unpublished data analysis tools. HW wrote the manuscript, with contributions from JL-P and NF and comments from all other authors.

## Conflict of Interest

The authors declare that the research was conducted in the absence of any commercial or financial relationships that could be construed as a potential conflict of interest.

## Footnotes

^1^https://github.com/PrincetonUniversity/princeton-mvpa-toolbox
